# Multifunctional Leather Surface Design by Using Carbon Nanotube-Based Composites

**DOI:** 10.3390/ma14113003

**Published:** 2021-06-01

**Authors:** Maria Stanca, Carmen Gaidau, Cosmin-Andrei Alexe, Ioana Stanculescu, Silvana Vasilca, Andreea Matei, Demetra Simion, Roxana-Rodica Constantinescu

**Affiliations:** 1 Research and Development National Institute for Textiles and Leather, Division Leather and Footwear Research Institute, Leather Research Department, 93, Ion Minulescu Str., 031215 Bucharest, Romania; maria.stanca@icpi.ro (M.S.); cosmin.alexe@icpi.ro (C.-A.A.); demetra.simion@icpi.ro (D.S.); rodica.constantinescu@icpi.ro (R.-R.C.); 2 Horia Hulubei National Institute for Physics and Nuclear Engineering, 30 Aleea Reactorului, 077125 Magurele, Ilfov, Romania; silvana.vasilca@nipne.ro or; 3 Department of Physical Chemistry, University of Bucharest, 4–12 Regina Elisabeta Bd., 030018 Bucharest, Romania; 4 Department of Analytical Chemistry Faculty of Chemistry, University of Bucharest, 90–92 Panduri Ave., 050067 Bucharest, Romania; 5 INFLPR–National Institute for Laser, Plasma and Radiation Physics, 077125 Magurele, Ilfov, Romania; andreea.purice@inflpr.ro

**Keywords:** conductive leather surface, multifunctional surface, CNT nanocomposites, leather surface, smart surface properties, antibacterial, self-cleaning

## Abstract

This paper deals with original research in smart leather surface design for the development of multifunctional properties by using multi-walled carbon nanotube (MWCNT)-based nanocomposites. The conductive properties were demonstrated for both sheepskin and bovine leather surfaces for 0.5% MWCNTs in finishing nanocompositions with prospects for new material design intended for flexible electronics or multifunctional leathers. The photocatalytic properties of bovine leather surface treated with 0.5% MWCNTs were shown against an olive oil stain after visible light exposure and were attributed to reactive oxygen species generation and supported by contact angle measurements in dynamic conditions. The volatile organic compounds’ decomposition and antibacterial tests confirmed the self-cleaning experimental conclusions. Ultraviolet protection factor had excellent values for leather surfaces treated with multi-walled carbon nanotube and the fastness resistance tests showed improved performance compared to control samples. Scanning electron microscopy with energy dispersive X-ray (SEM-EDX), X-ray photoelectron spectroscopy (XPS), and attenuated total reflection-Fourier transform infrared (ATR-FTIR) spectroscopy analysis confirmed the influence of different leather surfaces on MWCNT dispersion with an effect on nanoparticle reactivity and efficiency in self-cleaning properties. Multifunctional leather surfaces were designed and demonstrated through MWCNT-based nanocomposite use under conventional finishing conditions.

## 1. Introduction

The progress in carbon nanotube materials is in continuous development due to their large specific area, outstanding mechanical strength, fracture toughness, thermal stability, permittivity, and electrical conductivity with applications in electronics [1], protective clothing [2], biomaterials [3], or drug manufacture [4].

Inside the 2D hexagonal carbon nanotube lattice, the three electron valences are bonded to other carbon atoms while a single π electron shows very high mobility with quantum property in the 3D space and consequently outstanding conductivity, which has made these materials the most studied for electrical or electrochemical sensors [5].

Numerous research works for water pollutant removal such as Cr(VI) have been undertaken and demonstrated that at pH = 1, the redox reaction occurs with the formation of less toxic Cr(III), meanwhile at pH = 7, the absorption process takes place on the carbon nanotube multi-walled surface [6]. Compared to other classical carbon based materials, multi-walled carbon nanotube materials have shown a higher removing capacity of 200.0 to 1000.0 mg L^−1^ for Cr(VI). The functionalized or derivatized single-walled carbon nanotubes were prepared due to their less hydrophobic and aggregation ability in a water environment compared to the unfunctionalized materials. The photoreactivity of carboxylated single-walled carbon nanotubes in water and under visible light exposure was evaluated and showed evidence for the production of 1 O_2_ (singlet molecular oxygen), O_2_•^−^ (superoxide anions) and OH (hydroxyl radicals) [7]. The generation of 1O_2_ with oxidative properties for organic pollutants was reported as an induced reaction by UV exposure at 240 nm of multi-walled carbon nanotubes and attributed to the existence of defects in tube walls able to generate excited triple states [8]. The oxidant properties of microparticles functionalized with multi-walled carbon nanotubes were used for azo dye pollutant reduction in wastewater with 85% removal efficiency [9]. Other properties like antimicrobial, ultraviolet (UV), radio-frequency identification (RFID) and electromagnetic interference (EMI) blocking, wrinkle and heat resistance, optical display effect, etc. recommend carbon nanotube materials for functional product design [2].

Multifunctional textiles and leathers are known as the most promising materials for many applications like flexible electronics, smart automotive upholstery, professional or sports footwear, etc. Different nanocomposites have been used for the development of antimicrobial, self-cleaning, or flame-retardant properties on the leather surface by using silica [10,11,12,13,14], silver [15,16,17], zinc oxide [18,19,20,21], and titanium dioxide nanoparticles [22,23,24] as alternatives for the use of volatile organic compounds.

There are few approaches regarding the use of carbon-based nanomaterials or nanocomposites for leather surface finishing and performance improvement. Carboxylated graphene oxide (GO) nanoparticles were blended in waterborne polyacrylate composites and showed improved performances of leather surface finishing upon the dry and wet rubbing test and flexing endurance [25]. Maleic anhydride grafted with graphene oxide and polymerized with vinyl acetate was prepared and used for cattle leather retanning and showed good flame retardancy and UV light resistance, which was attributed to the graphene oxide influence and its UV radiation absorbing and scattering properties [26]. Nanocomposites based on polyurethane, carbon nanotubes (CNTs), and GO showed superior thermal, water, and mechanical resistance after experimental trials for dispersion improvement of CNTs [27]. The conversion of insulating properties of leather materials into conductive properties failed to be reached by using multi-walled carbon nanotubes due to their low dispersibility in water [28] and was successfully done by using polyaniline (PANI) and poly(3,4-ethylenedioxythiophene) (PEDOT), or by in situ polymerization of pyrrole [29,30]. Electrically conductive polymeric composites are a very challenging topic of research for electromagnetic radiation shielding and flexible electronics design and were recently achieved by using 25% sulfonated graphene oxide sheets blended in a water-based latex [31]. A recent review [32] on up-scaled functional leathers (UFL) underlines the high value utilization of a new generation of bio-based materials. In this new class of materials are included leathers with properties of absorbing electromagnetic waves and infrared light, X-ray shielding, electric conductive, flame-retardant, antibacterial, self-cleaning, water and oil repellent. The material used for successful electromagnetic radiation shielding was Cu@Ag nanoflakes, applied on the sheepskin leather surface after dispersion in ethanol and on a basecoat of poly(methyl methacrylate), which finally showed wearing resistance to 25,000 bendings [33]. Infrared radiation blocking leathers were developed by in situ creation of a 3D network of collagen fibers–SiO_2_ nanoparticles able to entrap air with a role in suppressing the nitrogen groups’ absorbing function, insulation, and reflecting infrared radiation. Infrared reflective leathers also showed multifunctional properties like flame retardancy and water repellency [34]. Bi/Ce nanoparticles, sodium tungstate, or bismuth iodide nanoparticles were used in leather retanning for X-ray reflective leathers. The new leathers with X-ray shielding properties were more comfortable and durable compared to traditional lead aprons [35]. Leather-based sensors showed good performance and can be included in the range of flexible electronics or electronic skins for health monitoring; moreover, they are very flexible, biocompatible, durable, and air permeable. The strain sensor was constructed based on leather treated with carbon black and covered with a conductive copper tape [36]. The same authors proved the ability of leather to be transformed in a wearable pressure sensor, leather based display, and user-interactive device after a treatment with a-CNTs and by inserting in systems based on dielectric coats, AgNWs or electroluminescence layer (ZnS:Cu) [37].

This paper deals with the application of multi-walled carbon nanotubes for the development of leather surface multifunctional properties. Compared to the state-of-the-art, we propose a facile method for polymer composite preparation and leather surface covering for the development of new properties: electric conductivity, UV radiation protection, rubbing and abrasion enhanced resistance, self-cleaning against oil soiling and volatile organic compounds, and antimicrobial resistance against *Escherichia coli* and *Staphylococcus aureus.*

The mechanism of multifunctional properties was evaluated on two kinds of leathers and was attributed to a well dispersed and optimum concentration of MWCNTs on the leather surface by integrating in film forming finishing composites with the efficient development of reactive oxygen species. In this regard, analyses were performed for oxygen and carbon energy binding by X-ray photoelectron spectroscopy (XPS), chemical functional group identification by attenuated total reflection-Fourier transform infrared (ATR-FTIR), structural morphology of leather coating by scanning electron microscopy (SEM) with energy dispersive X-ray analysis (EDX), dynamic contact angle measurements, and ultraviolet radiation transmitting properties. The results showed the potential for more durable leathers with multi protection functionalities to be produced by original and facile technology.

## 2. Materials and Methods

### 2.1. Preparation of Carbon Nanotube-Based Composites for Leather Surface Finishing

Multi-walled carbon nanotubes (MWCNTs) with O.D. × L of 6–9 nm × 5 μm, >95% carbon from Merck (Merck, Bucharest, Romania) were used in film forming composites for leather surface finishing. Compact acrylic-based film forming polymers, nitrocellulose based top finishing emulsions, and black pigment pastes were purchased from SC Triderma SRL (Bucharest, Romania).

Different concentrations of MWCNT, from 0.25% to 0.7% (% reported to film forming composite weight) were dispersed in acrylic and nitrocellulose-based polymers by using ultrasound stirring at 280 W and 50 Hz (ultrasonic bath, Elma S30H, Singen, Germany) for 15 min. The range of MWCNT concentrations was selected to avoid spray gun occlusion and to use the lowest quantity for maximum conductive effect without damaging the leather surface properties. Multifunctional properties were identified in order to add economic efficiency to the new approach. The trials for MWCNT pre-processing by ultrasound or ball-milling (not presented here) showed that the use of MWCNTs, as they are, is the most efficient route for the development of conductive and multifunctional properties.

### 2.2. Preparation of Leather Materials and Covering with Carbon Nanotube Composites

Bovine and sheepskin leather materials (SC Taro Comimpex srl, Jilava, Romania) were processed at the Leather Research Department in the crust stage for covering with new composites and classical composites in a black color.

**Figure 1:**
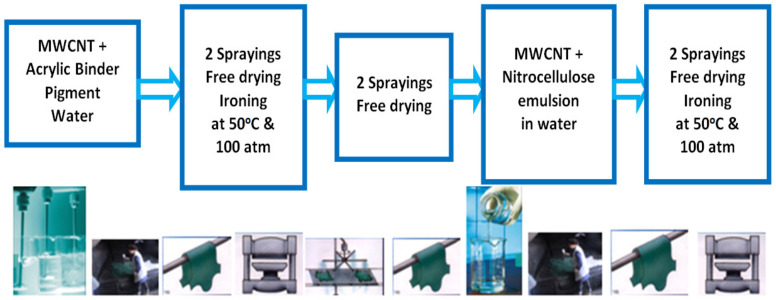
Leather finishing application technology [20].

The leather samples and control were processed with the classical finishing frame technology presented in Table 1 and Figure 1 by spraying layers of composites based on acrylic binders with pigmented black paste and a nitrocellulose emulsion for top fixing layers. The leather surface was covered with successive layers of base coat binders that were free dried and polymerized under heat and pressure according to classical technologies.

**Figure 2:**
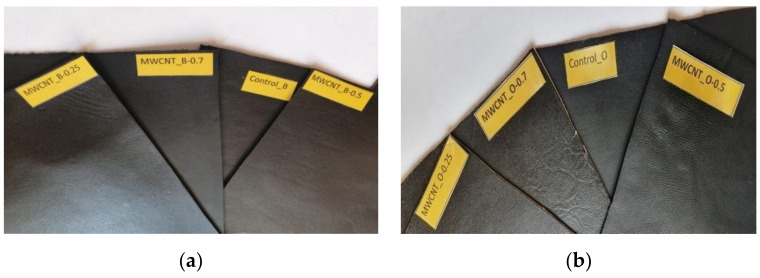
Bovine (**a**) and sheepskin (**b**) leathers treated with MWCNT-based composites.

The experiments were done on bovine and sheepskin crust leather surfaces with concentrations of MWCNTs of 0.25%, 0.5%, and 0.7% reported to finishing composite weight. Treated bovine leathers were encoded as MWCNT_B-0.25, MWCNT_B-0.50, MWCNT_B-0.70, and treated sheepskin leathers were encoded as MWCNT_O-0.25, MWCNT_O-0.50, MWCNT_O-0.70, while the control samples were Control_B and Control_O (Figure 2).

### 2.3. Characterization of Leather Surfaces

The main characteristics of new leathers were surface resistivity, photocatalytic properties (self-cleaning against olive oil stain and volatile compounds, antimicrobial resistance), ultraviolet protection factor, and physical–mechanical performances. Surface properties and the mechanism of self-cleaning effects were demonstrated by contact angle measurements in dynamic conditions, XPS, and ATR-FTIR spectroscopy as well as by analyzing the surface morphology and composition by SEM-EDX compared to the control samples. Ultraviolet protection factor measurements showed that the absorption properties of MWCNTs can recover and improve the genuine leather ultraviolet radiation protection. The results open the way for new smart materials, automotive upholstery leathers with multifunctional properties, and other innovative applications.

#### 2.3.1. Electric Surface Resistivity

**Figure 3:**
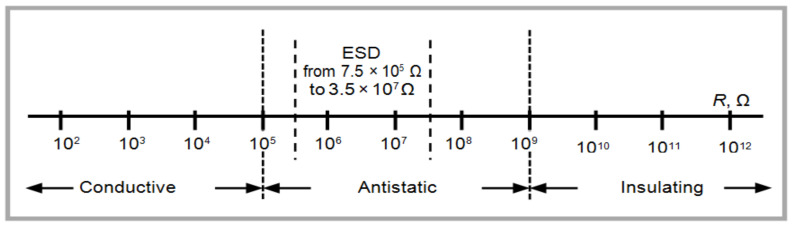
The scale of the electric properties of materials [39].

Leather is an insulating material, with electric resistivity value above 10^12^ Ω (Figure 3) and the conductive properties are important for many special fields like professional footwear, flexible electronics, touch screen gloves, healthy footwear, etc. Leather surfaces finished with composites based on multi-walled carbon nanotubes and control samples were characterized to determine electric surface resistivity properties (according to SR EN 1149-1 [38] by using an ohmmeter PRS 801 (Pro-Pack Materials Pte. Ltd., Singapore) with a potential difference of 100 ± 5 V for 15 ± 1 s). The results were reported as the average value of five determinations with a standard deviation below 5%.

#### 2.3.2. Ultraviolet Protection Factor

Ultraviolet protection factor (UPF) was calculated based on UV transmittance values measured with Jasco 400 UV–Vis spectrometer (Tokyo, Japan). The measurements of UV radiation transmittance were made in the range of a specific wavelength for UVA (320–400 nm) and UVB (290–320 nm). The calculation of UPF was done using Equation (1) [40]:

(1) $$\mathrm{UPF}=\frac{\mathrm{ED}}{{\mathrm{ED}}_{m}}=\frac{\sum_{290\;\mathrm{nm}}^{400\;\mathrm{nm}}E_{\lambda }S_{\lambda }\Delta_{\lambda }}{\sum_{290\;\mathrm{nm}}^{400\;\mathrm{nm}}E_{\lambda }S_{\lambda }T_{\lambda }\Delta_{\lambda }}$$

where E_λ_ = the erythemal spectral effectiveness; S_λ_ = solar spectral irradiance in W m^−2^ nm^−1^; T_λ_ = the spectral transmittance of the item; Δ_λ_ = the bandwidth in nm; and λ = the wavelength in nm.

The values were reported as the average of triplicate measurements with a standard deviation under 3%.

#### 2.3.3. Self-Cleaning Properties

The self-cleaning tests were performed against olive oil stains by exposing the leather surfaces to visible light using a UV–Vis Photoreactor, Labtech LIB-060M-PHR (Luzteh, Seoul, Korea). A drop of 5 μL of olive oil was placed on leather samples and control surfaces, then samples were exposed to visible light and surveyed up to 23 h.

#### 2.3.4. Contact Angle Measurement

The mechanism of self-cleaning properties was indirectly investigated by measuring the water drop contact angle in static and dynamic conditions by using aa VGA Optima XE Contact Angle Measurement device (AST Products, Inc., Billerica, MA, USA). The development of hydrophilic properties under Vis light exposure after 1, 2, 4, and 6 h were attributed to the formation of reactive oxygen species due to the specific surface chemistry and nanosize of MWCNTs in the interaction with water molecules from air [41].

#### 2.3.5. Volatile Organic Compound Analyses

Headspace gas chromatography (HS-GC) measurements were performed using a headspace sampler (7697A Agilent Technologies, Santa Clara, CA, USA) coupled with a gas chromatograph-mass spectrometer (GC6890N Agilent Technologies, Santa Clara, CA, USA). The GC separation was achieved using a capillary column HP-5MS (Agilent Technologies, Santa Clara, CA, USA). The operating conditions for GC were an initial temperature of 40 °C raised to 300 °C (10 °C/min) and the total run time was 40 min.

The operating conditions for headspace were sample equilibration temperature: 110 °C; sample loop temperature: 120 °C; transfer line temperature: 130 °C; and vial equilibration time: 40 min. The temperature for the transfer line was set at 280 °C, the electron impact (EI) energy was 70 eV, and the MS detector was operated in continuous scan mode with the m/z interval ranging from 10 to 700 amu.

In order to identify volatile organic compounds from leather, the samples were cut in squares and were introduced into headspace vials. The weight of the samples was 200 mg. Experimental data processing was performed with Agilent Enhanced ChemStation software (Agilent Technologies, Santa Clara, CA, USA) deconvolution of mass spectra with AMDIS software (NST, Gaithersburg, Maryland, USA), and confirmation of the molecular structure was conducted by intercomparison with the mass spectra of reference substances from the NIST 2005 library in NIST MS 2.0 software. (NST, Gaithersburg, Maryland, MD, USA) The semi-quantitative analysis was performed by normalizing the data to an external calibration mixture.

#### 2.3.6. Antimicrobial Properties of New Leather Surfaces Treated with MWCNTs

Antimicrobial surfaces are more and more important in everyday life and the use of non-active substances represents a stringent demand due to the pathogen increased resistance and toxicity of biocides. The antimicrobial properties of new leathers against *Escherichia coli* ATCC 25922 and *Staphylococcus aureus* ATCC 6538 (Mediclim, Otopeni, Romania) were assessed on non-sterile products by using the microbial numeration test following EN ISO 20743 [42]. The results were expressed as average values of triplicate samples.

#### 2.3.7. Physical–Mechanical Tests

Physical–mechanical tests were performed in order to evaluate the influence of MWCNTs on the surface properties related to water drop resistance according to STAS 82593 [43], abrasion resistance according to EN ISO 13520 [44], and rubbing fastness according to EN ISO 11640 [45]. The average of the physical–mechanical tests results were based on the values of three replicates.

#### 2.3.8. Scanning Electron Microscopy with Energy Dispersive X-ray Investigations

The identification of the carbon-based materials’ dispersion and aggregation on the leather surface was analyzed by SEM with a FEI Quanta 200 Scanning Electron Microscope (FEI, Eindhoven, The Netherlands) with a gaseous secondary electron GSED detector at an accelerating voltage of 12.5–20 kV. The energy-dispersive X-ray (EDX) unit was used for elemental analysis. The leather samples were coated with a thin Au layer of about 5 nm, in order to avoid charging effects. The MWCNT particle size distribution and average size were determined using OriginPro 7.5 (OriginLab, Northampton, MA, USA) with a manual measurement of 50 identified particles.

#### 2.3.9. X-ray Photoelectron Spectroscopy

X-ray photoelectron spectroscopy survey spectra and high-resolution XPS scan spectra were acquired using an Escalab Xi+ system, Thermo Scientific (Waltham, MA, USA). The survey scans were acquired using an Al Kα gun with a spot size of 500 µm, a pass energy of 50.0 eV, and an energy step size of 1.00 eV (10 scans). For the high-resolution XPS spectra, the pass energy was set to 10.0 eV, and the energy step size was 0.10 eV; 10 scans were accumulated for C1s and for O1s and 30 scans for N1s.

#### 2.3.10. Attenuated Total Reflection-Fourier Transform Infrared (ATR-FTIR) Spectroscopy

ATR-FTIR spectroscopy measurements were performed with a Bruker VERTEX 70 FTIR spectrometer (Bruker, Ettingen, Germany) equipped with a diamond crystal ATR unit in the 4000–600 cm^−1^ range with 64 scans and 4 cm^−1^ resolution. Spectra were measured against an air background and data were processed with OPUS 6.5 software (Bruker, Ettingen, Germany) using only atmospheric compensation. Triplicate measurements were performed to investigate the uniformity of applied leather finishing and to comparatively analyze its chemical composition taking into account the modifications of the bands’ position, intensity, and shape. Taking into account the similarity of the obtained spectra for both classical and MWCNT treatment, one may conclude that leather samples finishing showed excellent uniformity.

### 2.4. Statistical Analysis

The statistical processing was done using analysis of variance (ANOVA) (95% significant level) on each pair of interest and differences at *p* < 0.05 were considered statistically significant.

## 3. Results and Discussion

### 3.1. Surface Resistivity Measurements

Leathers are electrically insulating materials with a resistivity greater than 10^12^ Ω, as can be seen in Table 2. Surface resistivity measurement results showed that multi-walled carbon nanotubes are efficient for the development of electric conductivity properties on a leather surface (resistivity <10^4^ Ω). The resistivity measurements for classical bovine and sheepskin finished leathers showed high values for resistivity of 3.10 × 10^14^ Ω and 4.47 × 10^13^ Ω, respectively. We have to mention that the polymer layers for the control samples were performed as usual, but adapted to the need for surface covering, instead, the samples were coated with the same number of composite polymer layers with 0.25–0.7% MWCNTs in order to compare their influence on surface resistivity. The most conductive leather surfaces were recorded for the concentrations of 0.5% MWCNTs. Increased values for resistivity were recorded for higher concentrations of MWCNT, probably due to the difficulty of dispersion on the leather surface.

The transition of the acrylic polymeric composite from electrically insulating to conductive properties can be attributed to the outstanding conductivity of MWCNTs with a percolation threshold much lower than carbon black (conventional pigment) due to their extremely high aspect ratios. The percolation threshold of MWCNTs is estimated to be from 0.06 vol % to above 0.64 vol % in epoxy nanocomposites, depending on the dispersion state and aspect ratio preservation by different processing conditions [46]. The percolation thresholds of MWCNTs on the bovine leather surface was 0.002 vol % and on the sheepskin leather surface it was 0.001 vol %, lower compared to similar polymers filled with MWCNTs, which can be attributed to the combination with black carbon pigment and suitable dispersion [47]. In our experiments, the surface resistivity increased at a concentration of 0.7% MWCNTs, probably due to the nanoparticle agglomeration [47]. The capacitive touch screen sensitivity of new leather surfaces is presented in Supplementary Materials S2 compared to classical leathers. The results open the way to flexible and wearable electronics, the development of touch screen devices, and other applications like 5G electromagnetic interference shielding [31], etc. Compared to other achievements in the area, the conductive properties were developed by using low quantities of MWCNTs integrated in classical technologies of leather surface finishing without supplementary processing, excepting the ultrasound assisted dispersion of MWCNTs. Wegene and Thanikaivelan [29] succeeded in processing sheepskin leathers for gloves able to operate smart phones, tablets, and iPods by in situ double polymerization at 5 °C of 0.3 M pyrrole, 10% anthraquinone-2-sulfonic acid sodium salt monohydrate as the dopant, and 2.67 M ferric chloride as the oxidant. The same authors considered that the patented and the commercial methods for leather conductivity preparation based on leather surface finishing with polymeric composites and silver, gold, copper, carbon black, carbon nanotubes, graphite additives, or by embroidering of conductive metallic threads did not ensure even properties. The performance of patented leathers is limited to resistivity under 3.0 × 10^5^ –1.0 × 10^6^ Ω/sq, which may be the cause of inconstant electric conductivity.

### 3.2. Ultraviolet Protection Factor

It is already known that UV-protective garments including textile and leather clothes, hats, shoes, use in personal apparel, and sportswear are important for avoiding sunburns on short-time and cancer on long-time exposure. Ultraviolet protection factor (UPF) is the index for the protection of skin provided by clothes against ultraviolet radiation A (320–400 nm) and B (290–320 nm), responsible for immunological reactions and skin cancer. UV protective clothes have different degrees of protection according to their ability to block the transmittance of the radiation through skin by absorbing and/or reflecting it.

There is little information related to leather materials’ UPF because leathers have natural protective properties through a high density of collagen fibers. However, what is not known is whether the natural leather protection properties are worsened by the use of numerous auxiliary chemical materials. According to our measurements, unfinished leathers have UPF values of 19 for light colors, up to 45 for black colors, and by finishing, these values dropped to values of 2 and 3 (Table 3).

The results showed that leather surfaces treated with multi-walled carbon nanotubes have excellent ultraviolet protection factor for MWCNT_B-0.5, MWCNT_B-0.7, and MWCNT_O-0.5 compared with the control samples (Table 3). In the case of sheepskin leathers, the UV light absorbance was less intense, perhaps due to different surface grains compared to the bovine leather surface (Figure 2), which is characterized by many more wool follicles, and a smaller dermis thickness. As every graphene layer absorbs 2.3% of white light and reflects less than 0.1% [48], we can explain the contribution of MWCNTs to the recovery of the natural UV protection capacity of leathers, lost after the classical surface finishing. A high increase in the solar absorbance value of 90% was recently reported for polyester fabrics coated by the knife-over-roll technique with 5%, 10%, and 15% graphene dispersed in acrylic-based coating pastes [49].

From the results of electric resistivity and UPF values, we decided that the optimum concentration of MWCNTs for bovine and sheepskin leather surface finishing was 0.5% reported to the composite weight. Further investigations were focused on the MWCNT_B-0.5 and MWCNT_O-0.5 samples to identify other useful properties, which can add value to new leather surfaces in order to increase the economic efficiency by new functionalities.

### 3.3. Self-Cleaning Properties

**Figure 4:**
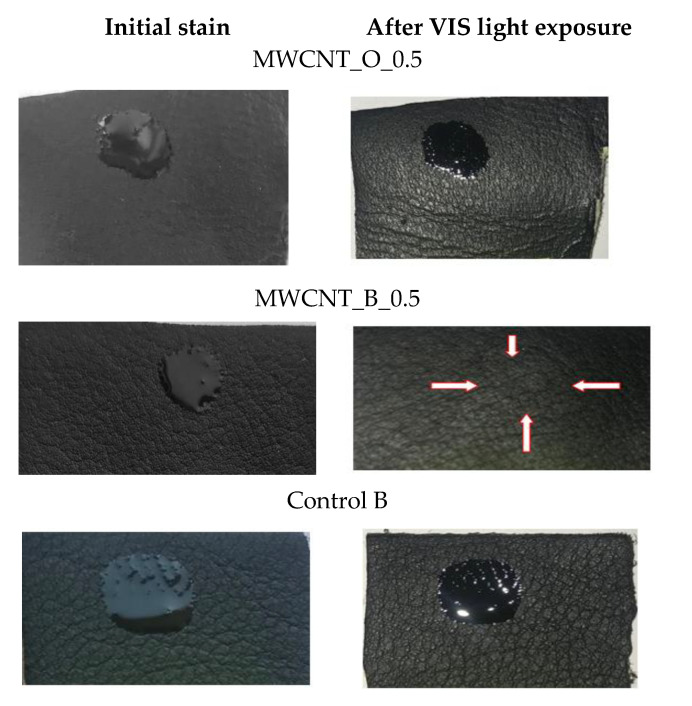
Photocatalytic decomposition of the olive oil stain on the bovine leather surface treated with 0.5% MWCNTs after 6 h of visible light exposure compared to the bovine control sample and MWCNT_O_0.5 sample, digital photos.

Leather durability is considered as an ecological treat that can prolong the life cycle and minimize the pollution. Leather items can be covered with soil resistant film forming polymers [50], can be dry-cleaned by using organic solvents, and can be restored by refreshing the initial finishing cover. All these actions are time, materials, and labor consuming, with a high ecological impact for the environment when organic solvents are used. In this context, photocatalytic nanomaterials with self-cleaning properties can contribute to leather item durability and consumer comfort. Some papers have investigated the self-cleaning properties of leather surfaces treated with nano TiO_2_ [23], and doped nano TiO_2_ [15]. In our present research, we performed some trials regarding the self-cleaning properties of leather surfaces against olive oil as a model for leather soiling with fatty substances. After the exposure of selected and control leather surface samples to visible light, we discovered that the olive oil stain was no longer visible after 6 h on the MWCNT_B-0.5 sample compared to the control and MWCNT_O-0.5 sample. In Figure 4, it can be seen that only the stain contour can be recognized compared to the other samples for which the olive oil drop was almost unchanged. The decomposition of the olive oil stain on sheepskin leather surface occurred after 22.5 h of visible light exposure for the MWCNT_O-0.5 sample. The same experiments performed on leather surfaces covered with 0.7% MWCNTs on both surface types (bovine and sheepskin) showed that the olive oil decomposed after 7 h and 12.5 h of visible light exposure, respectively (not showed here).

The decomposition of the olive oil stain occurred due to reactive oxygen species (O_2_•^−^, •OH, and H_2_O_2_) generated by MWCNTs on the leather surface under visible light exposure or in dark conditions [51]. The generation of reactive oxygen species by CNTs under sunlight exposure was confirmed by different studies [7] as well as the influence of reactive oxygen species (ROS) generated by MWCNTs on cell viability [52]. In order to understand if the mechanism of olive oil decomposition was due to the MWCNT photocatalytic activity under visible light exposure, further experiments on contact angle of water drop in dynamic conditions and antimicrobial resistance tests on leather surfaces were performed. Based on the same hypothesis of organic compound decomposition under photocatalytic mechanism induced by MWCNTs, the decomposition of volatile organic compounds under headspace conditions was performed, which is an important property for automotive leathers, contributing to suppressing the fogging process on car windscreens or on indoor air quality.

### 3.4. Contact Angle Measurements in Static and Dynamic Conditions

Static and dynamic contact angle of water drops were measured in the case of the initial prepared samples after 1 h, 2 h, 3 h, and 6 h of visible light exposure in order to identify the photocatalytic generated species with influence on the surface contact angle change.

**Figure 5:**
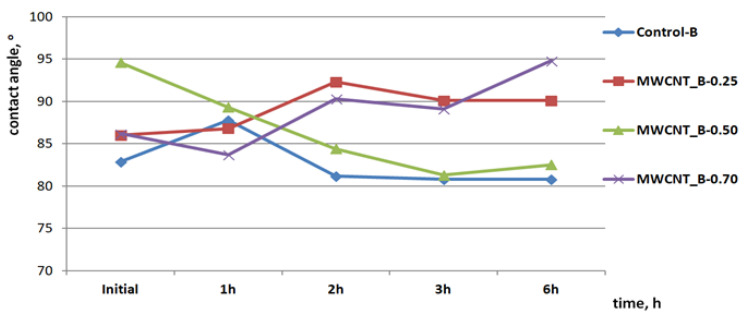
Static and dynamic contact angle of the water drop on the bovine leather surfaces covered with MWCNTs compared with the control sample.

**Figure 6:**
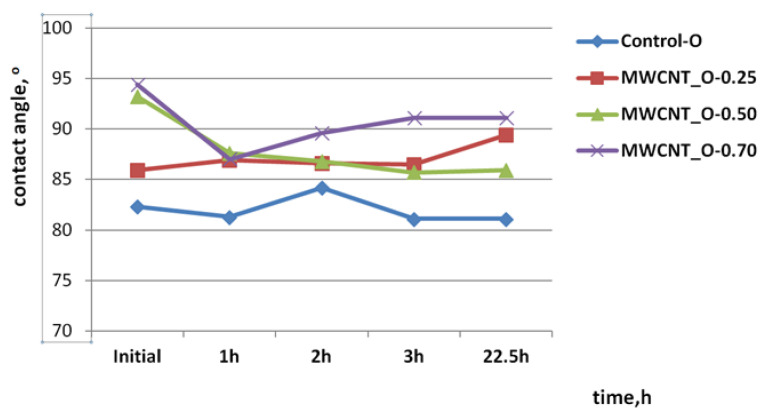
Static and dynamic contact angle of the water drop on the ovine leather surfaces covered with MWCNTs compared with the control sample.

The dynamic contact angle measurements for different concentrations of MWCNTs on bovine leather surfaces are presented in Figure 5 and showed a hydrophobic behavior when the water drop was in contact with the initial surfaces compared to the control surface. The hydrophobic properties increased after 1 h of visible light exposure for MWCNT_B-0.25 and the control sample, probably due to a dehydration process compared to the MWCNT_B-0.50 and MWCNT_B-0.70 samples for which the contact angle showed a more hydrophilic surface compared to the initial state. The contact angle dropped by 5.30° after 1 h of visible light exposure in the case of the leather surface covered with 0.5% MWCNTs compared with 2.50°, the difference in the contact angle of the MWCNT_B-0.70 sample and compared with the initial state. The contact angle of the water drop on the leather surface of the MWCNT_B-0.50 sample after visible light exposure showed a hydrophilic trend with a significant difference between the initial state and final value of 12.1° after 6 h of exposure, which is typical for photocatalytic surfaces with self-cleaning properties [14,15,23] and in obvious contrast with the other surfaces covered with different concentrations of MWCNTs (4.1° and 8.6°, respectively). This behavior can explain the decomposition of the olive oil stain on the leather surface covered with 0.5% MWCNTs compared to the other samples and with the control sample (2.1° contact angle difference after 6 h of visible light exposure). Sheepskin surfaces showed hydrophobic properties in static conditions and hydrophobic properties after visible light exposure with a slighter trend for hydrophilic properties of the MWCNT_O-0.5 sample by a 7.30° decrease in water drop contact angle after 22.5 h of visible light exposure (Figure 6). Compared to the control samples and other leather surfaces with contact angle differences of 1.2°, 3.5° and 3.3°, respectively, the MWCNT_O-0.5 sample showed the highest difference, in agreement with the photodecomposition performance, confirming the hypothesis of ROS generation. This behavior is in agreement with the literature findings and can explain the photocatalytic decomposition of the olive oil stain under visible light exposure.

### 3.5. Volatile Organic Compound Decomposition

The volatile organic compounds (VOC) released by the leather materials in different conditions are important in identifying the specific odor of leather natural products and mostly represent specific requirements for automotive industry related to the toxicological emitted substances or the ability to condensate on the inside of windshields. Odor and volatile organic compounds are mainly generated by natural or synthetic fat materials, vegetable tannins, or other auxiliary chemical products such as biocides, flame retardants, finishing solvents, or silicon based compounds, stabilizers used for hide and leather processing. In order to reduce the volatile organic compounds from leathers, a long list of processing steps and chemical material alternatives (tensides, enzymes, antioxidants, cross-linkers, calcium sequestrants, etc.) must be followed, all with an impact on the final product cost. The ability of nanomaterials to decompose the organic compounds under a photocatalytic oxidation mechanism has not yet been explored for volatile organic compounds generated by leather items.

**Figure 7:**
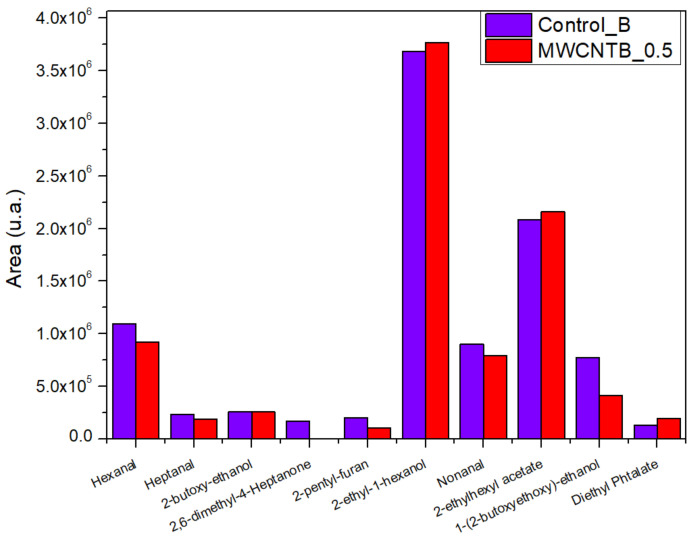
VOC released by bovine treated leathers compared with the control sample.

**Figure 8:**
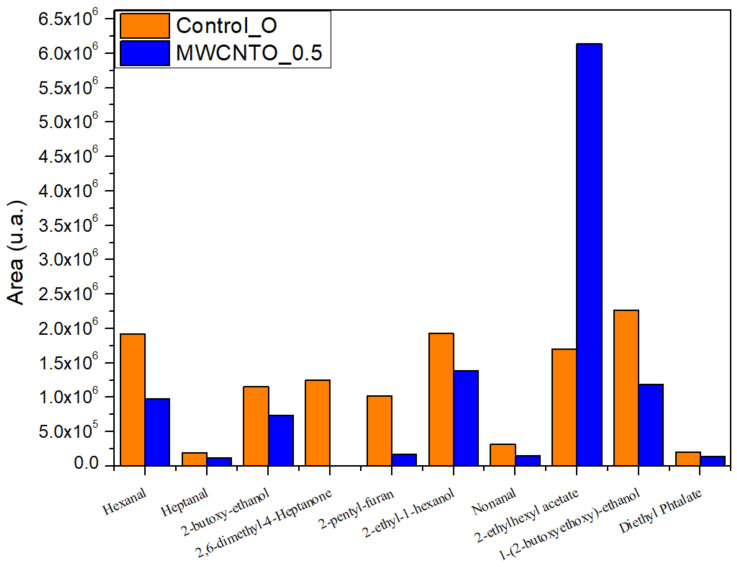
VOCs released by the sheepskin treated leathers compared with the control sample.

According to the measurements by HS-GC analysis, in all samples, we identified aldehydes (hexanal, heptanal, nonanal) and a heterocyclic organic compound (2-pentyl-furan), side products of natural fats originated from raw hides or fat liquoring materials; alcohols (2-butoxy-ethanol, 2-ethyl-1-hexanol, 1-(2-butoxyethoxy)-ethanol), an ester (2-ethylhexyl acetate), and a phthalate (diethyl phthalate) generated by finishing solvents and other additives like plasticizers [53]. Most of these compounds were found in lower quantities in MWCNTs treated bovine and sheepskin leathers compared to the control samples (Figure 7 and Figure 8). It was also found that the compound 2,6-dimethyl-4-heptanone, a specific volatile organic compound for the leather industry that can be found in wastewater treatment plants and is associated with leather odor [54], does not exist in MWCNTs treated bovine and sheepskin leathers. We assumed that because the ovine leathers were thinner than bovine leathers and their surface was larger for the same weight sample that fewer volatile compounds would be found in higher quantities compared to the control sample and bovine leathers (Figure 8). Further investigations will be performed in this promising area of research in light of the optimum time and temperature condition settings for a reduction in VOCs.

### 3.6. Antimicrobial Properties

The CNTs’ cytotoxicity was demonstrated in different experiments under sunlight or dark conditions [47] and was attributed to the generation of reactive oxygen species (^1^O_2_, O_2_•^−^, •OH, O_2_^−2^, OH•, etc.) with efficiency in damaging the DNA’s microorganisms.

It is already known that ROS are effective against most Gram-positive and Gram-negative organisms including antibiotic resistant microorganisms. From the most known ROS, the production of high levels of OH• may have the highest efficiency for antimicrobial-mediated lethality [55] The emergence of chemical biocides with high toxicity for humans and the environment has stimulated the interest in solid nanoparticles as alternatives to volatile organic antimicrobials. From Table 4 and Supplementary Materials S3, it can be seen that the surfaces covered with 0.5% MWCNTs showed antimicrobial properties and leather surfaces were disinfected or sterile with 99.7% and 100% reduction of *Staphylococcus aureus*, and with 100% and 99.4% reduction of *Escherichia coli*, respectively. The experimental results indicated that the well-designed application of MWCNTs embedded in polymer nanocomposites for leather surface finishing has the potential to improve health protection. The embedded nanoparticles in film forming polymers are less toxic compared to powder nanoparticles. The results are in correlation with the self-cleaning test, attributed to the photocatalytic mechanism. The studies on antimicrobial activity of MWCNTs revealed a lower cytotoxicity compared to SWCNTs due to a higher size diameter, which is a key factor for the mechanism based on the inhibition of the respiratory cell system. It was found that a good dispersion of MWCNTs and a concentration of 3.24 g/L could inhibit the microbial communities of cells from activated sludge by 80% [52,56] Compared to the reported concentrations, we found that 5 g/L of MWCNTs was well dispersed on the leather surface and were antimicrobial against Gram-positive and Gram-negative bacteria (Table 4). Other studies reported a mechanical mechanism of cell membrane and cytoplasm destruction of *Escherichia coli* bacteria in combination with oxidative stress based on ROS generation. Synergistic mechanisms of CNTs’ antimicrobial activity based on cell destruction and oxidative stress was recognized in many cases [53,57].

### 3.7. Physical-Mechanical Properties

From Table 5 and Table 6, it can be seen that for all samples treated with MWCNTs, the resistance to abrasion was improved compared to the control samples and all tests showed improved values for the selected leather surface samples, MWCNT_B-0.50 and MWCNT_O-0.50, respectively. The rubbing test results showed higher resistance of color on the leather surface (the higher mark means the higher resistance) and comparable results for color transfer on rubbing felt materials with the control samples (the second mark for the same test). The water drop test showed improved behavior for the sheepskin surface compared to the control sample (Table 6). It can be concluded that MWCNTs can add a higher resistance to abrasion, rubbing (dry, with water and perspiration), and improve dyeing fastness to the leather surface with an effect on leather item durability and increased consumer comfort. The potential of nanoparticle leachability and environmental pollution was also reduced. These results are in agreement with the research carried out on carboxylated graphene oxide modified waterborne polyacrylate finishing coatings [25].

### 3.8. Scanning Electron Microscopy with Energy Dispersive X-ray Investigation

**Figure 9:**
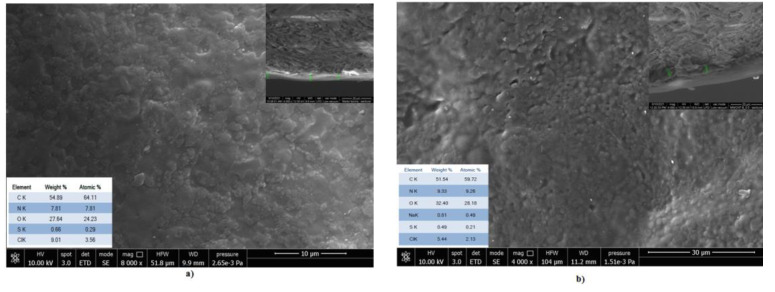
SEM images of bovine leathers (**a**) control and (**b**) MWCNT_B_0.5 for the surface with the inset images of cross sections and finishing layer measurement and elemental composition tables.

**Figure 10:**
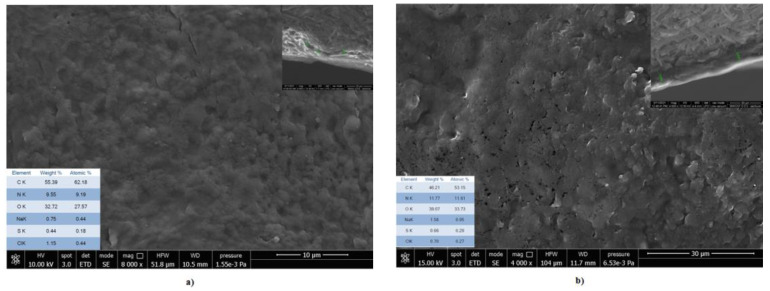
SEM images of ovine leathers (**a**) control and (**b**) MWCNT_O_0.5 for the surface with the inset images of cross sections and finishing layer measurement and elemental composition tables.

SEM images (Figure 9 and Figure 10) showed that the finishing composite with multi-walled carbon nanotubes was more uniformly distributed on the bovine leather surface and the thickness of the finishing coat increased compared to the control sample from 4.12–5.17 µm to 6.49–7.12 µm (inset images). Instead, the finishing coat of the sheepskin leathers increased from 4.01–6.13 µm to 10.07–10.71 µm compared to the control samples. Due to the different morphology of the sheepskin surface with numerous wool follicles and softer surface, the tendency of MWCNTs to agglomerate was obvious. The measurement of the MWCNT size confirmed the higher average size on the sheepskin leather surface (3.48 µm) compared to the bovine leather surface (2.53 µm). Supplementary Materials S4 presents the images of MWCNTs around the hair follicles and the distribution of MWCNTs on the leather surfaces. These results explain the more uniform distribution of MWCNTs on the bovine leather surface with improved performances (photocatalytic self-cleaning, UPF). The aggregation state of CNT materials is recognized as having a significant role in ^1^O_2_ production under light radiation or in a water environment [7]. The positive influence of shorter CNTs on the polymer composite conductivity was explained by the ratio of the size of the filler to matrix on low percolation threshold achievement [46].

EDX analyses confirmed a higher concentration of oxygen on the leather surfaces treated with MWCNTs compared to the classical surfaces (inset images), in agreement with XPS analyses. The presence of 4.15–9.0 wt% O in pristine MWCNT compositions was also reported [58,59].

### 3.9. X-ray Photoelectron Spectroscopy

**Figure 11:**
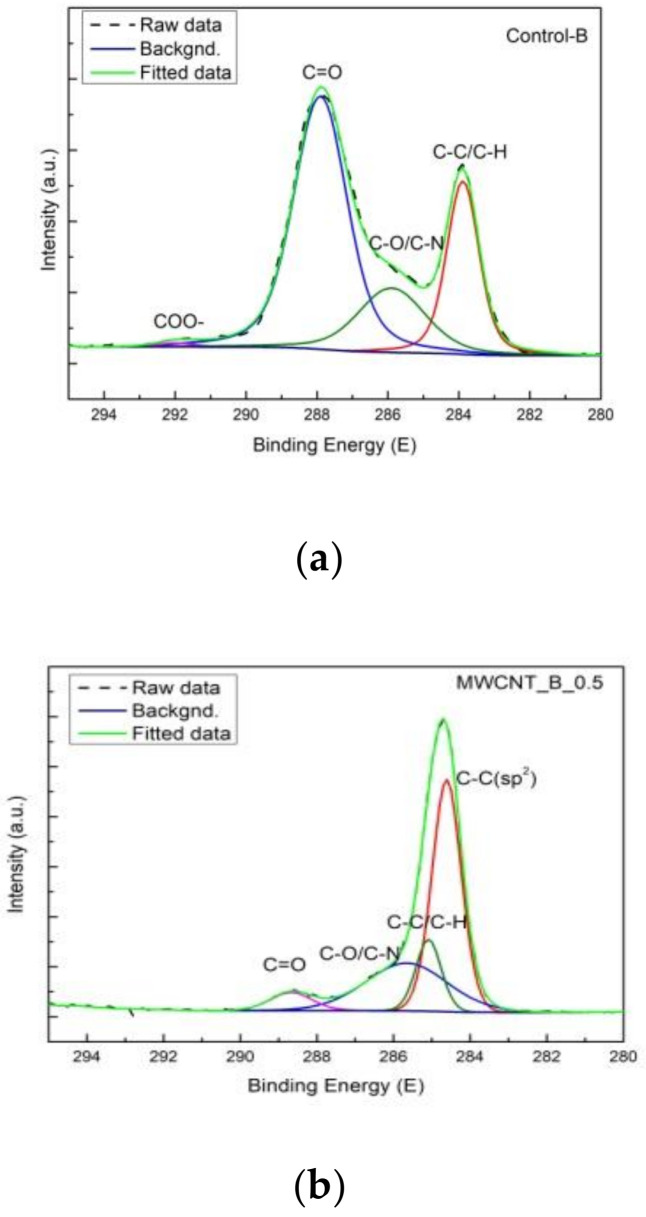
XPS spectrum of the (**a**) bovine control leather surface and (**b**) MWCNT_B_0.5.

XPS analysis was done to investigate the surface chemistry (i.e., C, N, O elements chemical states, binding energy) for the control and functionalized leather samples. Figure 11 shows the identified peaks for C1s corresponding to: C–C/C–N at 284.8 eV, C–O/C–N at about 286 eV, C=O at 288 eV with an additional peak only for MWCNT_B_0.5 at 284.4 eV, corresponding to C–C (sp^2^). The peak at about 290 eV, corresponding to COO- in the pristine sample disappeared for the treated sample. Regarding O1s binding energy, the peak at 535.17 eV, present in the control sample and attributed to C–O and C=O, disappeared for the MWCNT_B_0.5 sample. These differences suggest a potential interaction of carboxylic and carbonyl groups from the binder composite with MWCNTs on the leather surface.

**Figure 12:**
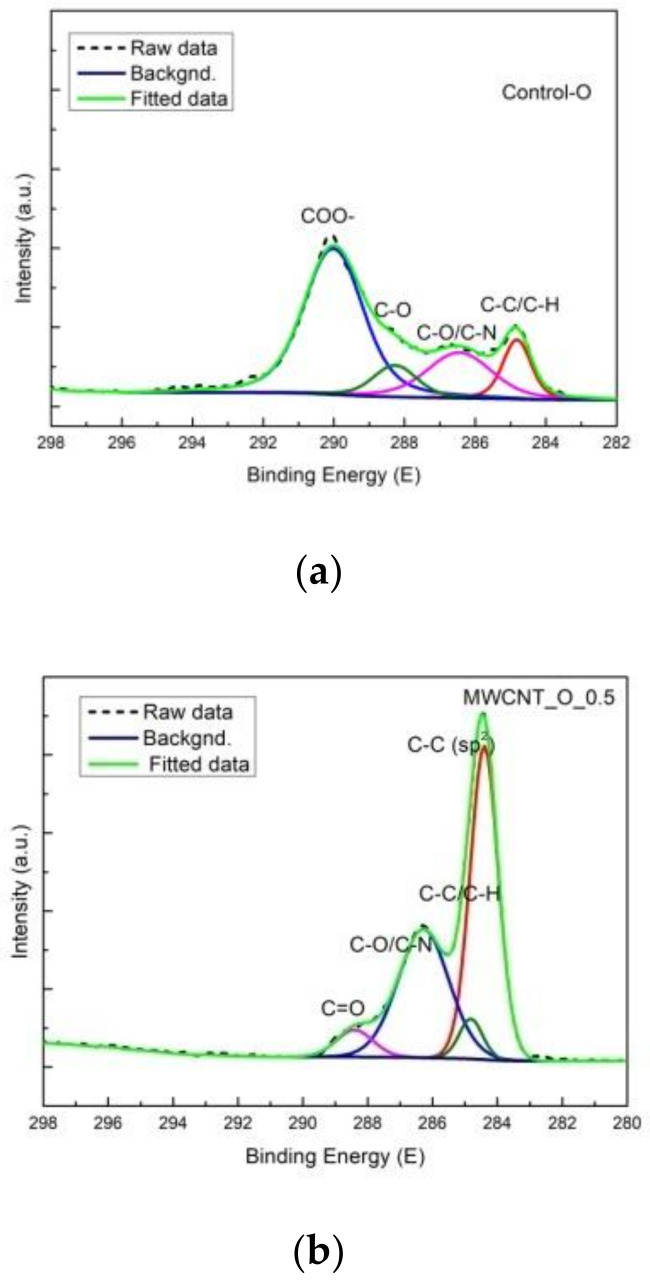
XPS spectrum of the (**a**) sheepskin control leather surface and (**b**) MWCNT_O_0.5.

In Figure 12, it can be seen that the identified peaks for C1s corresponded to: C–C/C–N at 284.8 eV, C–O/C–N at about 286 eV, C=O at 288 eV with an additional peak only for MWCNT_O_0.5, with significant increase at 284.2 eV, corresponding to C–C (sp^2^). The peak at about 290 eV, corresponding to COO- in the pristine sample disappeared for the treated sample. Regarding O1s binding energy, the peak at 535 eV, found in the control sample and attributed to C–O and C=O, disappeared for the MWCNT_O_0.5 sample. These differences suggest a potential interaction of carboxylic and carbonyl groups with MWCNTs on the leather surface. Taking into consideration the substantial increased concentration of O1s of 15.05% for the sheepskin leather surface compared to 4.51% for the bovine leather surface (Table 7), we can assume that the phenomena are connected to the more agglomerated MWCNT particles. Supplementary Materials S5 shows a binding energy of C1s and O1s for all of the investigated surfaces and of the MWCNT_O_0.25 sample with the elemental composition. An increased concentration of 21.54% O1s on the MWCNT_O_0.25 surface could be observed, which confirms the influence of MWCNTs on the finishing coat composition.

The increased concentration of oxygen on the MWCNT surface was attributed to the free electron interaction with carboxylic groups with an effect on reduced mobility [56] and lower performances in reactive species generation. The increased concentration of oxygen on the sheepskin surface can be attributed to MWCNT agglomeration and potential interaction with functional chemical groups of composite binders with an effect on lower performance in oil stain decomposition.

### 3.10. The Attenuated Total Reflection-Fourier Transform Infrared (*ATR*-*FTIR*) Spectroscopy

**Figure 13:**
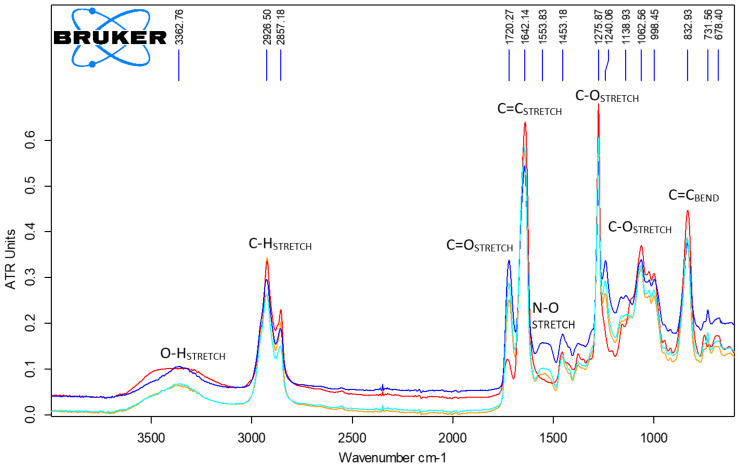
ATR spectra of control (**sheepskin** and **bovine**) and MWCNT 0.5 samples (**sheepskin** and **bovine**).

Figure 13 shows the best quality spectra of each control and functionalized sample. The main functional groups vibration modes: O–H, C–H, N–O, C–O, C=O, C=C stretching and C=C bending of nitrocellulose, acrylic, and carbon nanotube structures were identified, confirming the presence of the surface treatment [60].

The classical treatment applied to sheepskin and bovine leathers had an almost similar IR fingerprint while the MWCNT finishing composites induced differences in the ATR spectra. The influence of higher agglomerated MWCNT particles on the sheepskin leather surface on the binder polymers’ functional groups can be seen in the increase in intensity and broadening of the OH band (300–3500 cm^−1^), decrease in intensity of the C=O band at about 1720 cm^−1^, and the disappearance of the C–O stretch at 1240 cm^−1^, which is in agreement with significant modifications of binding energy found by XPS measurements. The spectra suggest a potential interaction of MWCNT particles with carboxylic and carbonylic groups of acrylic and nitrocellulosic binders, with a blocking effect on the free electron reactivity on the sheepskin surface and slight influence on the bovine leather surface due to the optimum dispersion level. These differences may explain the variation in the photocatalytic self-cleaning properties of the two kinds of leathers.

## 4. Conclusions

This paper deals with original research in smart leather surface design for the development of multifunctional properties. The conductive properties were demonstrated for both sheepskin and bovine leather surfaces for 0.5% MWCNTs in finishing nanocomposition with prospect for flexible electronics or new multifunctional leather material design. The photocatalytic properties of bovine leather surface treated with 0.5% MWCNTs were shown against an olive oil stain after visible light exposure and were attributed to reactive oxygen species generation and supported by contact angle measurements in dynamic conditions, with the generation of hydrophilic properties. The volatile organic compounds’ decomposition under head-space conditions and antibacterial tests confirmed the photocatalytic induced reactivity of MWCNTs dispersed in finishing composites. Ultraviolet protection factor had excellent values for leather surfaces treated with multi-walled carbon nanotubes due to the improved UV absorptive properties and fastness resistance tests showed also higher values compared to the control samples.

Scanning electron microscopy (SEM) coupled with electron dispersive diffraction (EDX), X-ray photoelectron spectroscopy (XPS), and attenuated total reflectance-Fourier transform infrared (ATR-FTIR) spectroscopy investigations revealed different distributions of MWCNTs on the leather surfaces, with an effect on the macroscopic level performances. Well distributed MWCNTs on the bovine leather surface due to the flat and even surface influenced the photocatalytic activity and UV absorbing properties compared to the sheepskin leather surface where more agglomerated particles and a higher concentration of oxygen was found.

The leather surface finishing followed conventional technologies with minimal modifications and the smart properties of MWCNT were revealed to be a result of the new film forming nanocomposite application.

## Figures and Tables

**Table 1:** Leather finishing technology for functionalization with MWCNTs.

Material: Bovine or Sheep Leather Surface Processed in Crust Stage		Finishing Technology			
Color: Black					
No	Materials for Leather Surface Finishing	Layer, mL/L			Application Layer 1: 2 × sprays, free drying, ironing at 50 °C and 100 kPa Layer 2: 2 × sprays, free drying Layer 3: 2 × sprays, free drying, ironing at 50 °C and 100 kPa
		1	2	3	
1	Compact acrylic binder, Pigment paste (Control and samples) + MWCNT (samples), Water	250 110 640			
2	Compact acrylic binder, Pigment paste (Control) + MWCNT (samples), Water		250 110 640		
3	Nitrocellulose based emulsion, Water (Control) + MWCNT (samples)			700 300	

**Table 2:** Surface resistivity of the untreated and treated leathers with MWCNTs.

Leather Sample	Resistivity, Ω/sq
Control_B	3.10 × 10^14^
MWCNT_B-0.25	4.00 × 10^3^
MWCNT_B-0.50	0.50 × 10^3^
MWCNT_B-0.70	1.20 × 10^3^
Control_O	4.47 × 10^13^
MWCNT_O-0.25	1.98 × 10^3^
MWCNT_O-0.50	2.00 × 10^3^
MWCNT_O-0.70	5.00 × 10^3^

**Table 3:** UPF of leather surfaces treated with multi-walled carbon nanotubes.

Leather Sample	UPF
Control_B	2
MWCNT_B-0.25	19
MWCNT_B-0.50	50
MWCNT_B-0.70	53
Control_O	3
MWCNT_O-0.25	13
MWCNT_O-0.50	40
MWCNT_O-0.70	19

**Table 4:** Antimicrobial properties of bovine and sheepskin leathers treated with MWCNTs.

Sample	Result Inoculums Concentration, CFU/mL	Reduction, %
*Staphylococcus aureus* ATCC 6538	T0 = 3.95 × 10^3^	–
Control_O	T24 = 4.75 × 10^2^	87.97
Control_B	T24 = 1.91 × 10	95.16
MWCNT_O-0.5	T24 = 1.2 × 10	99.70
MWCNT_B-0.50	T24 = 0	100
*Escherichia coli* ATCC 25922	T0 = 4.72 × 10^3^	–
Control_O	T24 = 4.33 × 10^2^	90.83
Control_B	T24 = 2.76 × 10^2^	94.15
MWCNT_O-0.50	T24 = 0	100
MWCNT_B-0.50	T24 = 2.8 × 10	99.41

**Table 5:** Physical–mechanical resistance of the bovine leather surface treated with MWCNTs.

Characteristics	Sample			
Dyeing Fastness to Rubbing Test:	Control-B	MWCNT_B-0.25	MWCNT_B-0.50	MWCNT_B-0.70
100 dry cycles, Marks	5/4–5	5/4–5	5/4–5	4–5/4–5
50 wet cycles, Marks	3–4/3	5/2	5/3–4	5/2
50 perspiration solution cycles, pH = 8, Marks	4/3–4	4–5/1	5/2–3	4–5/1
Water drop resistance, Marks	5	4–5	5	4–5
Abrasion resistance, No of revolutions	Cracking at 38,400 revolutions	Withstands 51,200 revolutions	Withstands 51,200 revolutions	Withstands 51,200 revolutions

**Table 6:** Physical–mechanical resistance of the sheepskin leather surface treated with MWCNTs.

Characteristics	Sample			
Dyeing Fastness to Rubbing Test:	Control-O	MWCNT_O-0.25	MWCNT_O-0.50	MWCNT_O-0.70
100 dry cycles, Marks	5/4–5	5/4–5	4–5/4	5/4–5
50 wet cycles, Marks	1/1–2	5/2	4–5/1–2	5/2
50 perspiration solution cycles, pH = 8, Marks	1/1	4–5/1	4/1	4–5/1
Water drop resistance, Marks	4	5	5	5
Abrasion resistance, No of revolutions	Cracking at 12,800 revolutions	Withstands 51,200 revolutions	Withstands 51,200 revolutions	Withstands 51,200 revolutions

**Table 7:** C, O, and N atomic concentration on the leather surface.

Name	Control-B	MWCNT_B_0.5	Control-O	MWCNT_O_0.5
	Atomic %		Atomic %	
C1s	84.71	79.25	81.67	66.54
O1s	14.13	18.64	17.13	32.18
N1s	1.16	2.11	1.19	1.29

## Data Availability

Data sharing is not applicable to this article.

## Supplementary Materials

The following are available online at https://www.mdpi.com/article/10.3390/ma14113003/s1, S1: Percolation thresholds for leather surfaces, S2: Video demonstration for touch screen properties of leathers covered with MWCNT nanocomposites as effect of electric conductivity new properties development as compared to classical treated leathers, S3: Antimicrobial properties of selected samples as compared to control samples, S4: SEM images of MWCNT particles around the hair/wool follicles and the distribution of MWCNT size on leather surfaces, S5: XPS survey for C1s and O1s for MWCNT_B_0.5, MWCNT_O_0.5, MWCNT_O_0.25, Control-B, Control-O and Elemental composition of MWCNT_O_0.25.
