# Supplementary material for: Multifunctional Leather Surface Design by Using Carbon Nanotube-Based Composites
Source: Materials (Basel). 2021 Jun 1;14(11):3003. doi: 10.3390/ma14113003 (PMC8199507; doi:10.3390/ma14113003)
Supplement: Supplementary file 1 [file materials-14-03003-s001.zip › materials-1204523-supplementary/checked-Supplementary materials.pdf]

materials

MDPI

Supplementary materials

Multifunctional Leather Surface Design by Using Carbon Nanotube-Based Composites

Maria Stanca [1], Carmen Gaidau [1,*], Cosmin-Andrei Alexe [1], Ioana Stanculescu [2,3,*], Silvana Vasilca [2,4], Andreea Matei [5], Demetra Simion [1] and Roxana-Rodica Constantinescu[1]

1 Research and Development National Institute for Textiles and Leather, Division Leather and Footwear Research Institute, Leather Research Department, 93, Ion Minulescu Str., 031215 Bucharest, Romania; maria.stanca@icpi.ro (M.S.); cosmin.alexe@icpi.ro (C.-A.A.); demetra.simion@icpi.ro (D.S.), rodica.constantinescu@icpi.ro (R.-R.C.)
2 Horia Hulubei National Institute for Physics and Nuclear Engineering, 30 Aleea Reactorului, 077125 Magurele, Ilfov, Romania; silvana.vasilca@nipne.ro
3 Department of Physical Chemistry, University of Bucharest, 4–12 Regina Elisabeta Bd., 030018 Bucharest, Romania
4 Department of Analytical Chemistry Faculty of Chemistry, University of Bucharest, 90–92 Panduri Ave., 050067 Bucharest, Romania; silvana.vasilca@unibuc.ro
5 INFLPR–National Institute for Laser, Plasma and Radiation Physics, RO-077125 Magurele, Romania; andreea.purice@inflpr.ro
* Correspondence: carmen.gaidau@icpi.ro (C.G.); istanculescu@nipne.ro (I.S.)

S1. Percolation thresholds for leather surfaces

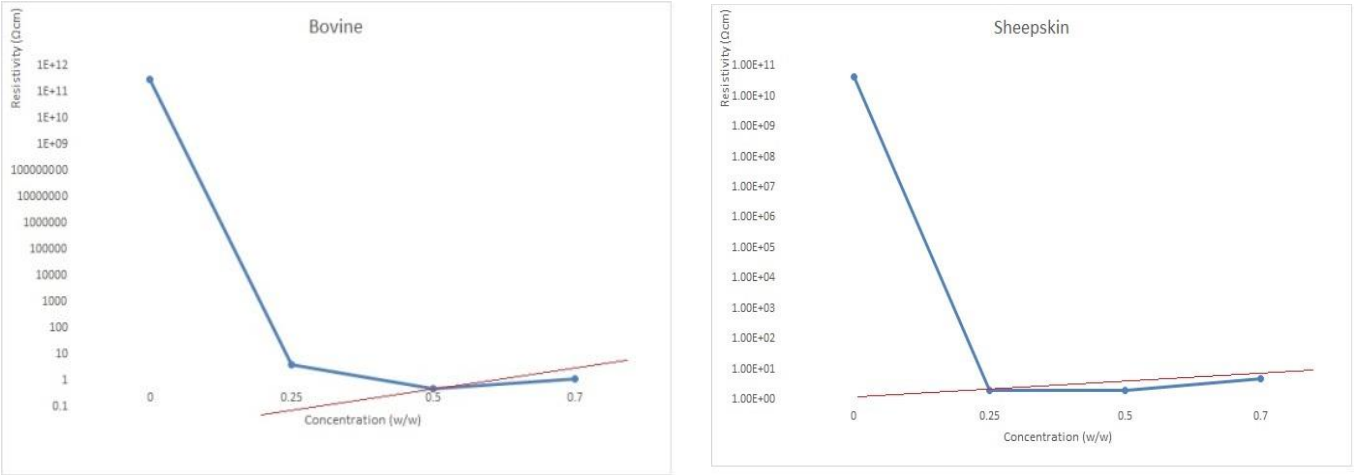

(**a**) (**b**)

Figure S1. The concentration of MWCNT corresponding to percolation thresholds for finishing composites:.

(**a**) bovine leather-1phr (0.002 vol % MWCNT) and (**b**) sheepskin leather-0.5 phr (0.001 vol % MWCNT).

S2. Video demonstration for touch screen properties of leathers covered with MWCNT nanocomposites as effect of electric conductivity new properties development as compared to classical treated leathers.

S3. Antimicrobial properties of selected samples as compared to control samples

Table S1. *Escherichia coli* ATCC 25922.

Sample	Inoculum concentration, CFU/mL	Photo	Reduction,%	Log_{10} red.
Initial concentration of inoculum	T_0=4.72x10^3	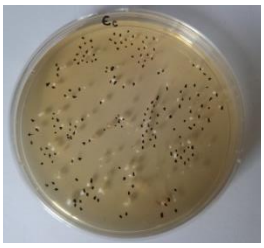	-	-
Control-O	T_{24}=4.33x10^2	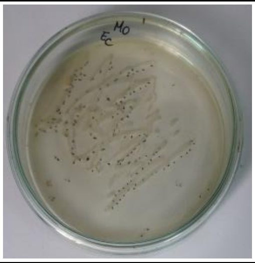	90.83	1.04
Control-B	T_{24}=2.76x10^2	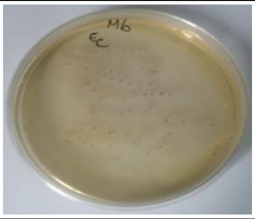	94.15	1.23
MWCNT_O-0.5	T_{24}=0	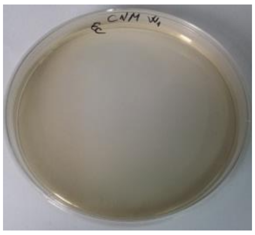	100	3.67
MWCNT_B-0.5	T_{24}=2.8x10	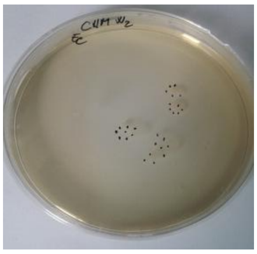	99.41	2.23

Table S2. Staphylococcus aureus ATCC 6538.

Sample	Inoculum concentration, CFU/mL	Photo	Reduction,%	Log_{10} red.
Initial concentration of inoculum	T_0=3.95x10^3	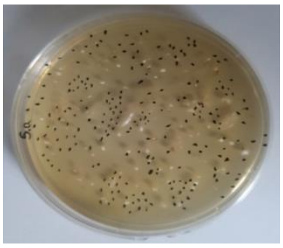	-	-

Control-O	T_{24}=4.75x10^{2}	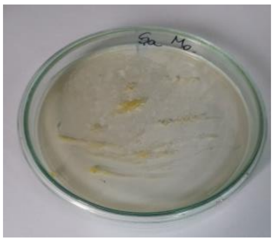	87.97	0.92
Control-B	T_{24}=1.91x10^{1}	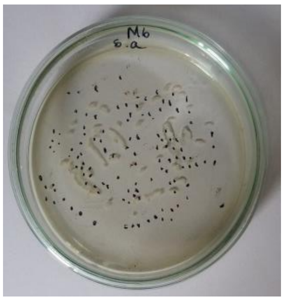	95.16	1.32
MWCNT_O-0.5	T_{24}=1.2 x10	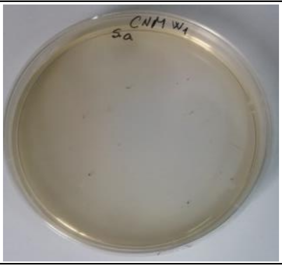	99.70	2.52
MWCNT_B-0.5	T_{24}=0	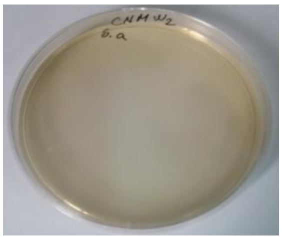	100	3.6

S4. SEM images of MWCNT particles around the hair/wool follicles and the distribution of MWCNT size on leather surfaces

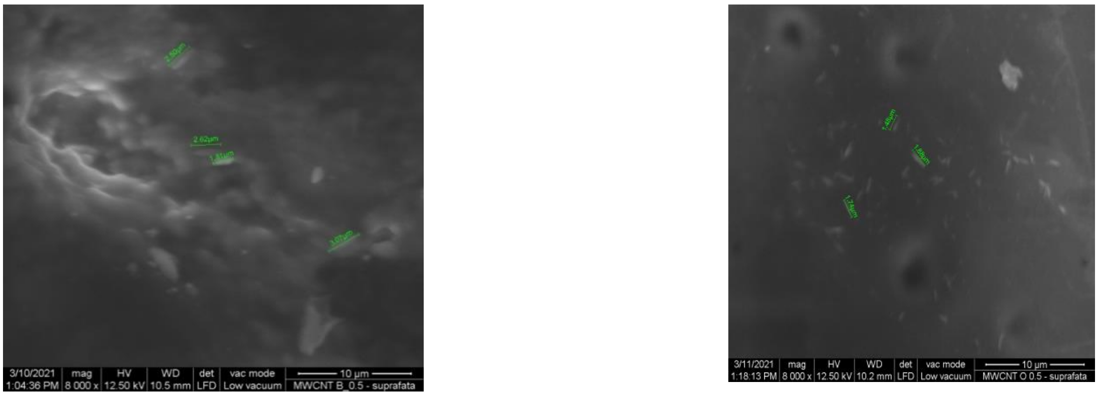

(**a**) (**b**)

Figure S2. SEM image of leather surfaces (**a**) MWCNT_B-0.5 and (**b**) MWCNT_O-0.5.

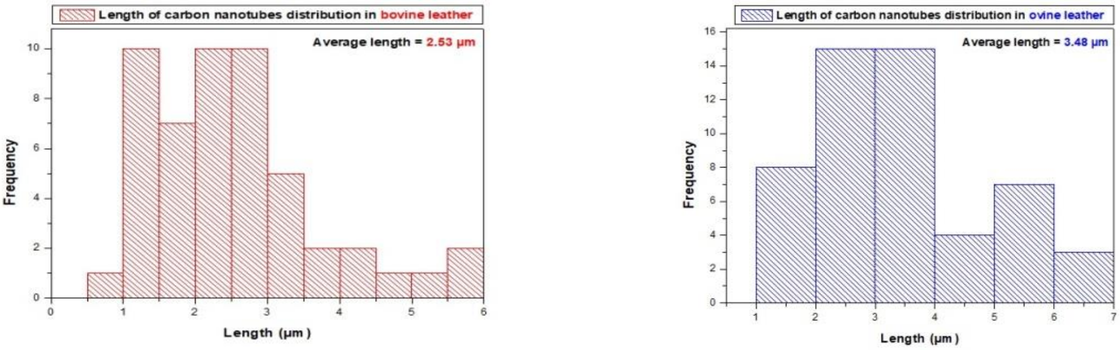

(**a**) (**b**)

Figure S3. MWCNT size distribution and average size particle on (**a**) bovine and (**b**) sheepskin leather surfaces.

S5 XPS survey for C1s and O1s for MWCNT_B_0.5, MWCNT_O_0.5, MWCNT_O_0.25, Control-B, Control-O and elemental composition of MWCNT_O_0.25

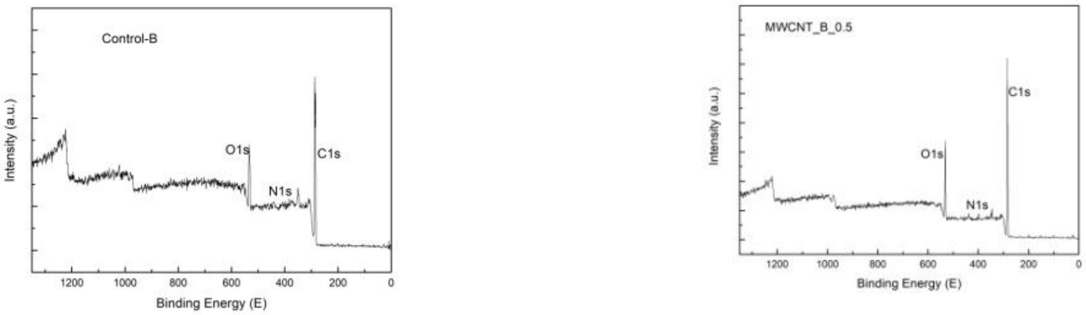

(**a**) (**b**)

Figure S4. XPS survey for C1s and O1s for bovine leather surfaces: (**a**) Control-B and (**b**) MWCNT_B_0.5.

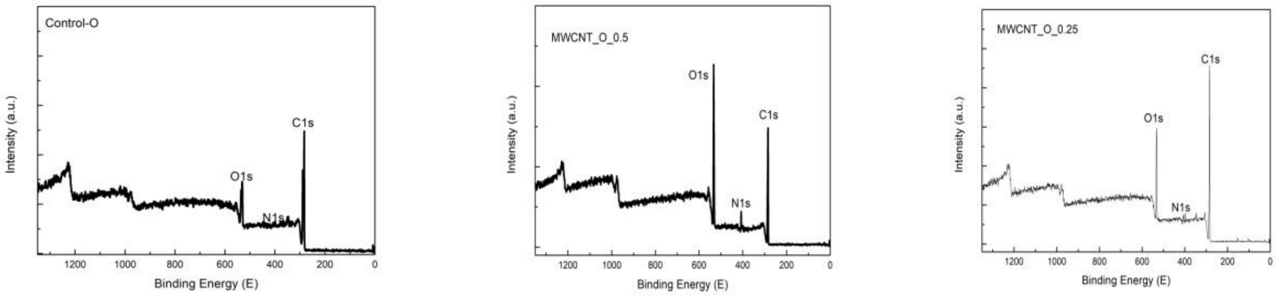

(**a**) (**b**) (**c**)

Figure S5. XPS survey for C1s and O1s for ovine leather surfaces: (**a**) Control-O, (**b**) MWCNT_O_0.25, (**c**) MWCNT_O_0.5.

Table S3. Elemental composition of MWCNT_O_0.25 sample.

Name	***Atomic %***
C1s	75.90
O1s	21.54
N1s	2.55
